# A differential privacy protecting K-means clustering algorithm based on contour coefficients

**DOI:** 10.1371/journal.pone.0206832

**Published:** 2018-11-21

**Authors:** Yaling Zhang, Na Liu, Shangping Wang

**Affiliations:** 1 School of Computer Science and Engineering, Xi’an University of Technology, Xi’an, ShaanXi, China; 2 School of Science, Xi’an University of Technology, Xi’an, ShaanXi, China; Institute of Computing and Information Technology, PAKISTAN

## Abstract

This paper, based on differential privacy protecting K-means clustering algorithm, realizes privacy protection by adding data-disturbing Laplace noise to cluster center point. In order to solve the problem of Laplace noise randomness which causes the center point to deviate, especially when poor availability of clustering results appears because of small privacy budget parameters, an improved differential privacy protecting K-means clustering algorithm was raised in this paper. The improved algorithm uses the contour coefficients to quantitatively evaluate the clustering effect of each iteration and add different noise to different clusters. In order to be adapted to the huge number of data, this paper provides an algorithm design in MapReduce Framework. Experimental finding shows that the new algorithm improves the availability of the algorithm clustering results under the condition of ensuring individual privacy without significantly increasing its operating time.

## Introduction

As an important access to information under the current big data environment, data mining is able to obtain useful information through statistics, machine learning, pattern recognition and other methods. The information obtained is widely used in business management, production control, market analysis and scientific research. Clustering analysis is a typical method of data mining, whose main idea is to group data, forming the biggest differences between various clusters and the smallest within every cluster. K-means is a simple clustering algorithm with high clustering speed that is adopted in various fields.

Clustering with multiview data is becoming a hot topic in data mining, pattern recognition, and machine learning. Documentary [1] presented a convex formulation of multi-view subspace learning that enforces conditional independence while reducing dimensionality. Documentary [2] addressed the problem of unsupervised clustering with multi-view data of high dimensionality, which proposed a new algorithm which learns discriminative subspaces in an unsupervised fashion based upon the assumption that a reliable clustering should assign same-class samples to the same cluster in each view. In documentary [3], basic multiview fuzzy clustering algorithm, called collaborative fuzzy c-means (Co-FCM), is firstly proposed. The algorithm settled two issues in multiview clustering, namely, how to combine the clustering result from each view and how to identify the importance of each view.

Under the background of big data, privacy disclosure of sensitive information has become a serious hurdle for the application of data mining. Differential privacy protecting is an attacking technique raised by Dwork for the first time in 2006.It adapts to any attacking technique under any background knowledge, so it has attracted a lot of attention for never being limited by the size of data sets. In K-means clustering analysis, differential privacy protecting technique can effectively reduce the exposure of individual privacy. The research on differential privacy protecting algorithm is of great significance.

Differential privacy protection is a data distortion technique. As for differential privacy protecting K-means clustering algorithm, it is necessary to study increasing the availability of clustering results while avoiding data exposure. Many research have been done by scholars from home and abroad. Documentary [4] proposed the issue of balance between availability and privacy of differential privacy protection. As differential privacy protection is a data distortion technique, the balance between availability and privacy of differential privacy protection is an NP problem. As for the effect of privacy budget *ε* on the balance between availability and privacy of differential privacy protection, documentary [5] proposed a new attack model to determine the value of the parameter, analyzed the model in detail and figured out a parameter selection formula through the theory and the model. Documentary [6] proposed a differential privacy protecting K-means clustering algorithm by which an improved method for the initial center point is proposed for the problem that the new center point is far from the original center point after the random noise is added considering the sensitivity of the initial center point. The improved method divides the dataset into subsets on average, and calculates the centers of each subset which are later set as the original center point in order to improve the accuracy of clustering and meanwhile it kept the premise of noise adding amount and privacy protection level unchanged. Documentary [7] proposed a K-means clustering method to support differential privacy under MapReduce framework which on the basis of adding differential privacy, calculates the distance of each record to the cluster center with the function mapping of MapReduce. The most time-consuming part of each iteration round is handled by the distributed computing resources, effectively improving the efficiency of K-means algorithm. However, data features are not considered in the algorithms above. Their research findings show high availability only when privacy budget *ε* is high. When privacy budget is low, no ideal availability is achieved. Documentary [8] suggests that differential privacy should be added to recommended system, thus noise should be added according to the level of systems. Privacy levels and interference ranges are randomly selected from a fixed level of privacy. An outlier-eliminated differential privacy (OEDP) k-means algorithm is proposed in documentary[9], in which the initial center points is selected in accordance with the distribution density of data points, and Laplacian noise is added to the original data for privacy preservation. Documentary [10] proposed a novel DPLK-means algorithm based on differential privacy, which improves the selection of the initial center points through performing the differential privacy Kmeans algorithm to each subset divided by the original dataset. Documentary [11] proposed a privacy and availability data clustering scheme (PADC), which enhances the selection of the initial center points and the distance calculation method from other points to center point.

However, clustering effect of different clusters in the same iteration is not taken into consideration by differential privacy protecting K-means clustering algorithms above. As a result, the same noise was added to different clusters, which may cause large deviation from the center point and low availability of clustering results. Based on the findings above, one of the main ideas of this paper is to add different noises to different clusters in each iteration to avoid too much random noise added to clustering sets of small size or large density. This will result in great deviation of center points, poor clustering effects and availability. Based on the application of the clustering analysis of big data, this paper proposed an algorithm under the MapReduce framework. The main contributions of this paper are as follows:

1. In order to increase the usability of clustering result when privacy budget is low, a new privacy budget allocation method is proposed based on the contour coefficients of each cluster. So, a new differential privacy protecting K-means clustering algorithm is designed. The analysis on the algorithm and the experiment result show that the new algorithm meets the requirement of differential privacy protection, and usability of clustering result is increased especially for the situation that privacy budget is low.
2. The algorithm in this paper is designed on the basis of MapReduce distributed environment to fit the need of application of big data. And the efficiency of algorithm is tested on multiple data sets, the experiment result show that the new differential privacy protecting K-means clustering algorithm can provide the higher usability of clustering result and the higher level of privacy protection and acceptable efficiency for multiple data sets.

## Relative basis

### Differential privacy protection

Differential privacy protection model is a privacy protection technology based on data distortion. By adding noise to distort data, it makes sure that the data privacy is under protection and meanwhile the data keeps its function for the data mining later.

**Definition 1** *ε*−Differential Privacy [12] assume there is random algorithm M and P_M_ is the collection of all possible output of M. As for any two neighboring data set D, D’ and S_M_, any subset of P_M_, if M fits the requirement below:

$$P_{r}\left[M\left(D\right)\in S_{M}\right]\leq exp(\varepsilon )\times Pr[M\left(D'\right)\in S_{M}]$$ (1)

M fits the requirement of *ε*−Differential Privacy Protection.

D and D’ are two neighboring subsets between which the difference is no more than one record. *ε* is a specified constant and is called privacy protection budget[13]. It’s easy to tell that as long as *ε* is small enough, attackers can hardly tell with the same output S_M,_ whether the query function functions on D or D’. When *ε* is 0, it can meet the requirement of the function only when all the output is noise. The query results can not reflect the characteristics of data, which means that *ε* is meaningful when it is larger than 0. Meanwhile, the smaller *ε* is, the better privacy is protected.

As for numeric query function, Laplace distribution mechanism is adopted in most cases. The return value of query function q, which functions on any dataset D, is *q*(*D*)+*x*. *q*(*D*) is the true value of the query function and *x* is a random value fitting Laplace distribution mechanism.

**Definition 2** Global sensitivity [14] Suppose there is a query function *f*:*D*→*R*^*d*^. When a dataset is input into it, the output *d* is a real number vector. As for any two neighboring data set D and D’,

$$\Delta f=\underset{D,D^{'}}{\max}\|f(D)-f(D^{'}){\|}_{1}$$ (2)

is called the global sensitivity of function *f*. Besides, ‖ ‖_1_ represents the sum of the absolute values of the vector’s elements.

**Definition 3** Laplace mechanism [14] Given a dataset *D*, suppose there is a function *f*:*D*→*R*^*d*^ and the sensitivity is Δ*f*, random algorithm *M*(*D*) = *f*(*D*)+*Y* provides *ε*−Differential Privacy Protection. Noise *Y* fits the Laplace distribution of Δ*f*/*ε*.

Laplace mechanism, by adding random noise which fits the Laplace distribution, to specific results, realizes differential privacy protection. When the location parameter of the Laplace distribution is 0 and the scale parameter of it is *b*, the Laplace distribution is recorded as *Lap*(*b*), and the probability density function is

$$p(x)=\frac{1}{2b}\exp(-\frac{\left|x\right|}{b})$$ (3)

It is easy to tell from the characteristics of Laplace distribution that the smaller *ε* is, the larger the random noise is.

Besides, sequence combination and parallel combination of privacy budgets play an important role in privacy distribution process of clustering algorithms [15].

**Characteristic 1 Sequence composition.** Suppose there are algorithms *M*_1_,*M*_2_…*M*_*n*_, and there privacy budgets are *ε*_1_,*ε*_2_…*ε*_*n*_. As for the same dataset *D*, *M*(*M*_1_(*D*),*M*_2_(*D*)…*M*_*n*_(*D*)), combination algorithms of {*M*_1_,*M*_2_…*M*_*n*_} on *D*, provides *ε*−differential privacy and $\varepsilon =\sum_{i=1}^{n}\varepsilon_{i}$.

**Characteristic 2 Parallel combination.** Suppose there are random algorithms *M*_1_,*M*_2_…*M*_*n*_, and there privacy budgets are *ε*_1_,*ε*_2_…*ε*_*n*_. Dividing *D* into disjoint datasets *D*_1_,*D*_2_…*D*_*n*_, combination algorithm *M*(*M*_1_(*D*),*M*_2_(*D*)…*M*_*n*_(*D*)) of algorithm {*M*_1_,*M*_2_…*M*_*n*_} provides *ε*−differential privacy and *ε* = max(*ε*_*i*_).

### Introduction and analysis of DP-Kmeans Algorithm

#### Main idea of DP-Kmeans algorithm

DP-Kmeans Algorithm [7] is a clustering algorithm which adds differential privacy protection to K-Means algorithm under distributed environment. Its main steps are:

**Step 1**: All records in the dataset are normalized, and the average distribution method is used to determine the initial cluster centers.

**Step 2**: The data records are equally divided into data pieces of the same size, and the Map operation and the Reduce operation are performed to obtain *num*, the number of the records of the same cluster and *sum*, the sum of attribute vectors of all the records in the cluster.

**Step 3**:Random noise of the same size is added to *num* and *sum* and the cluster center are calculated.

**Step 4**: Calculate whether the distance between the K cluster centers in the current round and the previous one is smaller than the given threshold. If it is, the algorithm is terminated, and output the number of clustering centers and clustering records. Otherwise, repeat steps 2 to 4.

#### Analysis of the characteristics of DP-Kmeans Algorithm

**Fig 1:**
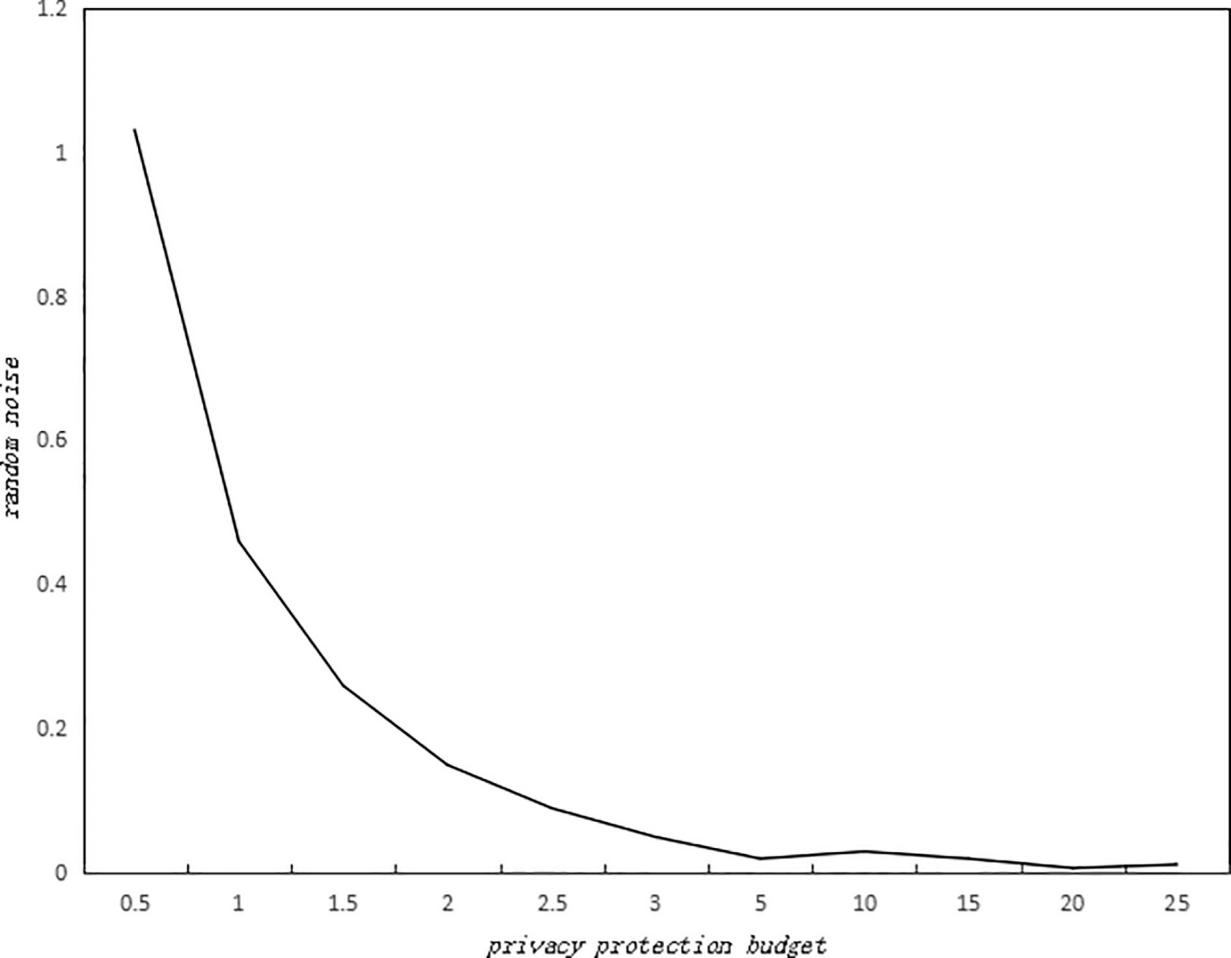
Effect of privacy budget on random noise.

According to the characteristics of Laplace Differential Privacy Protection Mechanism, the smaller *ε* is, the larger the random noise is. As for clustering algorithm, it can be told from its iteration nature and sequence combination of privacy budgets that when there are more iterations and smaller privacy budget, there is larger random noise. Considering the randomness of Laplace noise, this paper calculated the average value of 10 experiments. In the experiments, we suppose the global sensitivity is 1, and got the change of random noise for different privacy budgets (Fig 1). As is shown in Fig 1, the smaller the privacy protection budget, the greater the random noise, which means there is stronger privacy protection; the larger the privacy protection budget, the smaller the random noise, which means there is weaker privacy protection.

**Fig 2:**
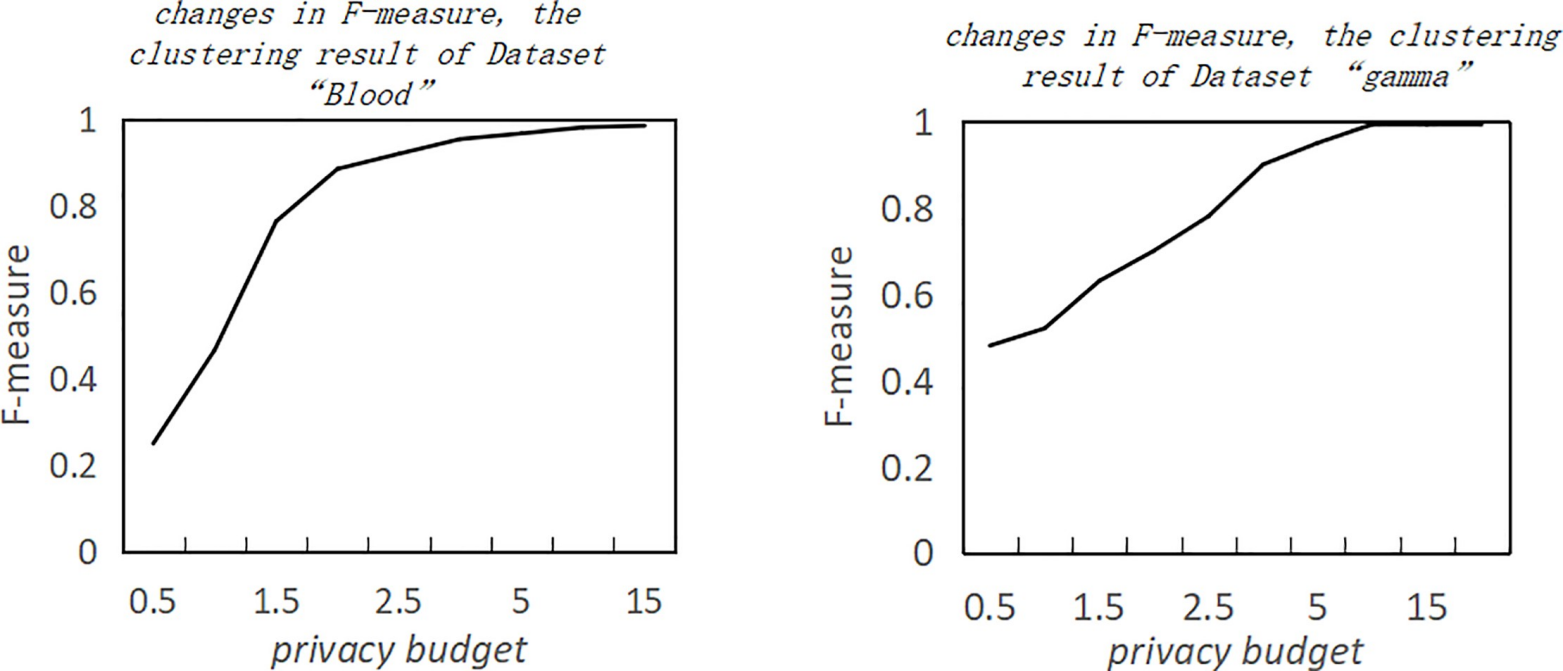
Effect of privacy budget on the functions of different dataset.

In Documentary [7], K-means clustering algorithm, an algorithm supporting differential privacy protection under MapReduce framework, adds the same random noise to each cluster center after each iteration of clustering with a distributed computing method. A distributed framework is used to improve the efficiency of the implementation of the algorithm. However, by observing the improved effect of the initial k-means algorithm in Documentary [7], we can easily tell from in Fig 2 that when the privacy budget is higher than 3, the algorithm provides higher availability in clustering results. When the privacy budget is low, the algorithm provides lower availability in clustering results. After thorough analysis, this paper found that when privacy budget is low, the added noise is large. It is possible that the noise will shift the cluster center, which may result in an increase in the number of clustering iterations and a decrease in the availability of clustering results.

As a matter of fact, a smaller privacy budget means stronger privacy protection. Increasing the data availability when privacy budget is low, is of great research significance. Maintaining a steady availability of clustering results with strong privacy protection(i.e. *ε* is low)is the focus of this paper. By assigning different clusters to different privacy budgets, this paper tries to avoid the problem of large deviations of cluster centers caused by data perturbations to increase the availability of clustering results and maintain strong privacy protection.

## DPK-means algorithm based on contour coefficients under MapReduce framework

According to the allocation strategy of privacy budget *ε*[16], when the number of iterations is large, the noise disturbance will increase significantly, and cause great impact on the result because of the uncertainty of the initial center and the number of iterations. As for the problem of low data availability with small privacy budget *ε*, this paper proposed a differential privacy protecting K-means clustering algorithm based on contour coefficients. The main idea of this algorithm is to evaluate the effect of clustering in each iteration with contour coefficients and to add different noise to clustering centers of each iteration according to the contour coefficients in order to solve the problem of low clustering availability caused by large deviations of cluster centers.

### Contour coefficients

#### Basic definition

contour coefficients is a way of evaluating clustering results. The combination of cohesion and resolution can be used to evaluate the effects of different algorithms or clustering results of different operation modes based on the same original data. As for the same sample point *i*, the contour coefficient calculation formula is as follows:

$$S(i)=\frac{bi-ai}{\max(ai,bi)}$$ (4)

In the formula, *ai* represents the average similarity between sample *i* and other samples in the same cluster. The smaller *ai* is, more sample *i* should be clustered. *bi* represents the minimum value of the average distance from *i* to all samples from other clusters. That is to say, *bi* = min{*bi*_1_,*bi*_2_,…,*bi*_*k*_}. The contour coefficient is in [–1,1]. The larger *S*(*i*) is, the closer the cluster where the point *i* locates is. So the average contour coefficient for each cluster is calculated as follows:

$$S(k)=\sum_{i=1}^{num_{k}}S(i)/num_{k}$$ (5)

In the formula, *num*_*k*_ stands for the number of samples in cluster No. k. The larger the *S*(*k*) value, the better the clustering effect and vice versa.

#### Improvement of the calculation of contour coefficient

The complexity of calculating the contour coefficient is *O*(*n*^2^). When the number of data increases, the computing time of the algorithm will grow rapidly. When the amount of data increases to a certain extent, it’s impossible to estimate the amount of computation. Even though the algorithm is under the MapReduce framework, the problem of too long algorithm running time when the data volume is large is not solved. The key point of contour coefficient is to calculate the cluster dissimilarity *ai* and inter-cluster dissimilarity *bi*. It is found from calculation that the time complexity of the contour coefficients can be reduced to *O*(*n*).

Suppose the records in cluster No. k are {*a*_1_,*a*_2_,…,*a*_*n*_} and the record dimension is d. The sum of attribute vectors recorded at the cluster center point is *sum* and the number of records is *num*. Then the dimensional value of clustering center is $(\sum_{i=0}^{n}a_{i})/n$ and the distance between every record in the cluster and the center is *ai*. The calculation formula is $\sqrt{\sum_{j=0}^{d}(\frac{\sum_{i=0}^{n}a_{i}^{d}}{n}-a_{k}^{d}{)}^{2}}$ which can be simplified into $\frac{\sum_{i=0}^{n}\sqrt{\sum_{j=0}^{d}{\left(a_{i}^{d}-a_{k}^{d}\right)}^{2}}}{n}$.

In contour coefficient *a*_*i*_ is calculated by the average distance between the center and the records in the same clusters. It is $\frac{\sum_{i=0}^{n}\sqrt{\sum_{j=0}^{d}{\left(a_{i}^{d}-a_{k}^{d}\right)}^{2}}}{n}$.

In conclusion, contour coefficient *a*_*i*_ is calculated by the average distances between the center and the records in the same clusters and *bi* is calculated by the distances between the records and centers of different clusters. In this way, time complexity is reduced from *O*(*n*^2^) to *O*(*n*).

### Description of the algorithm

Similar to Documentary [7], the algorithm in this paper is designed on the basis of MapReduce distributed environment in which dataset is divided into M pieces of the same size and the Map task and the Reduce task are executed on them respectively. Suppose that the dataset is *D*, the total number of records is *N*, the records are {*a*_1_,*a*_2_,…,*a*_*n*_}, the dimension of records is *d*, the center is recorded as *u*_*k*_, the privacy budget is *ε*, *t* is the number of iterations and the random noise of iteration *t* is $Noise_{k}^{t}$.

**Input:** dataset *D* and the number of clusters *K*.

**Output:** clustering sets fitting the requirement of differential privacy protection

**End condition:** the distance between the centers of two neighboring iterations is lower than one or the number of iterations is higher than 10.

**Fig 3:**
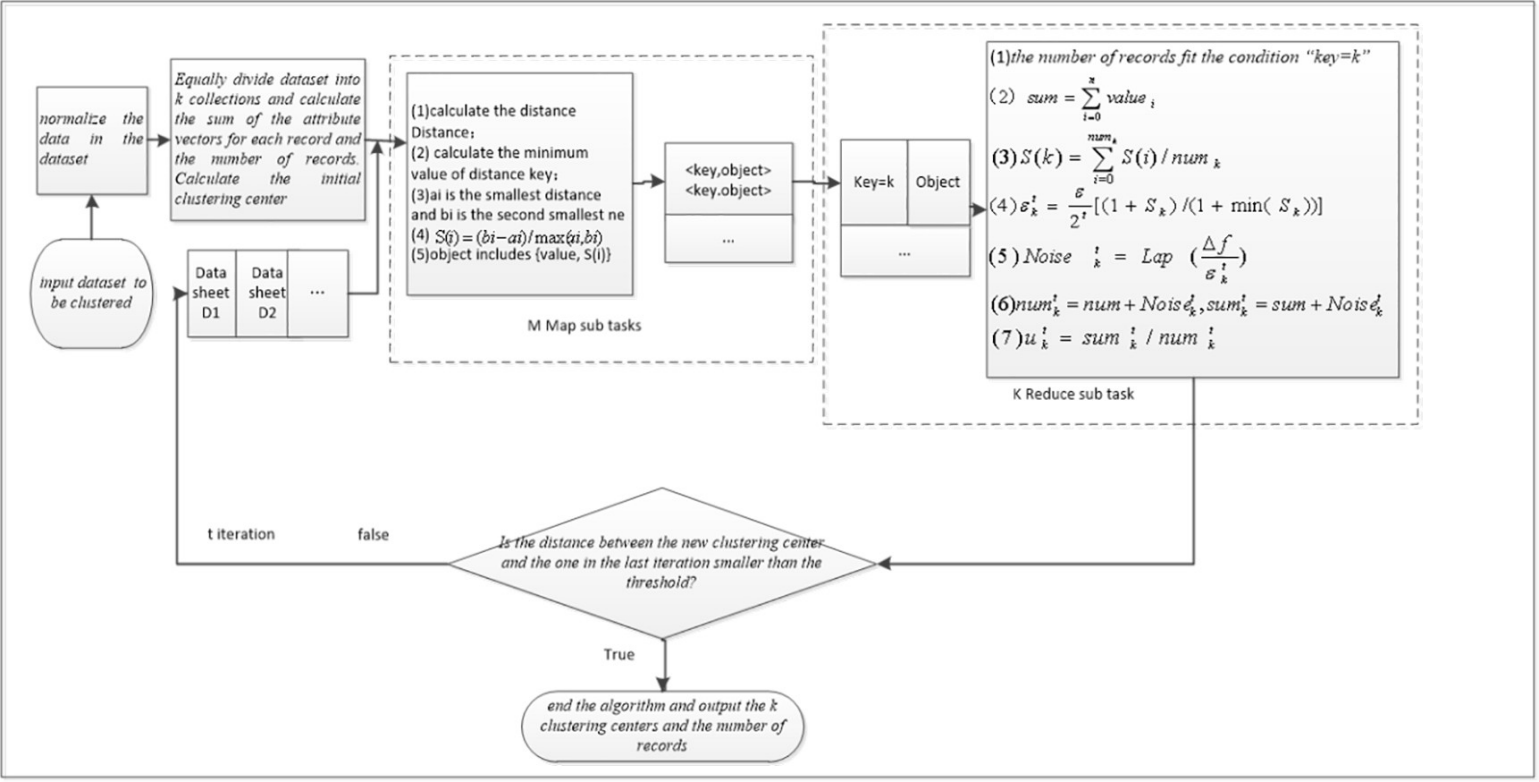
Flow chart of DPK-Means clustering algorithm based on contour coefficients.

1. All the data in the dataset *D* are normalized to make sure that all points are located in [0,1]^*d*^.
2. Equally divide dataset *D* with *N* records into *K* sets, namely *C*_1_,*C*_2_…*C*_*k*_. There are *N*/*K* records in Set *C*_*k*_.
3. Calculate $sum_{k}^{0}$, the sum of the attribute vectors for each record and $num_{k}^{0}$, the number of records in dataset *C*_*k*_. Add random noise $Noise_{k}^{0}$ to $sum_{k}^{0}$ and $num_{k}^{0}$ to get $sum_{k'}^{0}$ and $sum_{k'}^{0}$. Calculate the initial center point $u_{k}^{0}$, $u_{k}^{0}=sum_{k'}^{0}/num_{k'}^{0}$.
4. The main task divides all data records into *M* pieces, and assigns *M* sub-missions to implement the Map operation, and *K* sub-missions to implement the Reduce operations. Map sub-mission is operated on *N*/*M* records and calculate the distance from every record *a*_*i*_ to *k* clustering centers *u*_*k*_. And record the minimum value *u*_*k*_. The results are output in the form of <*key*,*value*>.
5. Reduce sub-mission is operated on all the <*key*,*value*> couples in the same clustering center and record *num*, the number of record in this clustering and *sum*, sum of the attribute vectors for each record. As for subset No. *K*, calculate *num*_*k*_, the number of record in this clustering and *sum*_*k*_, sum of the attribute vectors for each record.
6. Calculate contour coefficient *S*_*k*_ of *k* clusters and add random noise $Noise_{k}^{t}$ to *num*_*k*_ and *sum*_*k*_. As for the *k* clusters *S*_*k*_, find out the minimum value min*S*_*k*_. The privacy budget of cluster No. *k* in iteration No. *t* is $\varepsilon_{k}^{t}=\frac{\varepsilon }{2^{t}}[(1+S_{k})/(1+\min S_{k})]$. Random noise $Noise_{k}^{t}=Lap\left(\frac{\Delta f}{\varepsilon_{k}^{t}}\right)$.
7. Calculate the new clustering center $u_{k}=\left(sum_{k}+Noise_{k}^{t}\right)/\left(num_{k}+Noise_{k}^{t}\right)$.
8. Calculate the distance between the new clustering center and the one in the last iteration. If it is lower than the threshold, the algorithm ends and the clustering set is output. Otherwise, go back to step 4. The algorithm flow chart is shown in Fig 3.

## Analysis on the algorithm

### The privacy analysis

It is concluded from the chart that the privacy of K-means algorithm is operated by adding random Laplace noise to *sum*_*k*_, the sum of all the record vectors and *num*_*k*_, the number of records. As is known from Documentary [13] and Characteristic 1—-Sequence composition, in K-means algorithm, when the number of iterations is t, the privacy budget of every iteration is $\frac{\varepsilon }{t}$. When the number of iteration is uncertain, each iteration costs half of the privacy budget *ε*, which means the budget of t iterations is $\varepsilon =\sum_{t=1}^{T}\left(\frac{\varepsilon }{2^{t}}\right)$. In the formula, T represents the number of iterations. In the algorithm proposed in this paper, the number of iterations is uncertain, so the privacy budget is calculated in the second way above. According to Definition 1, the smaller *ε* is, the better the privacy is protected. According to Characteristic 2, to make sure that Iteration No. t fits the requirement of *ε*_*t*_−differential privacy, the privacy budget of the added random noise should be no more than $\frac{\varepsilon }{2^{t}}$. The algorithm above evaluates the clusters by the contour coefficients and adds noise accordingly. That is to say, small noise is added to clusters with better clustering effect and larger noise is added to clusters with worse clustering effect. The privacy budget of iteration No. t distributed by clustering center is $\varepsilon_{k}^{t}=\frac{\varepsilon }{2^{t}}[(1+S_{k})/(1+\min S_{k})]$. As *S*_*k*_ is in [–1,1], *S*_*k*_ apparently $\varepsilon_{k}^{t}\leq \frac{\varepsilon }{2^{t}}$.

According to Definition 2, the global sensitivity is the maximum difference between 2 neighboring datasets. In K-means clustering algorithm, the process of counting the number of clusters is like a counting function. The largest variation is 1 which means Δ*f*_*num*_ = 1. As for D dimensional space [0,1]^*d*^, the largest variation of the sum of all characteristics is 1 and the dimension of points is *d*. The global sensitivity of *sum*, namely Δ*f*_*sum*_ = *d*; the global sensitivity of the whole query sequence is Δ*f* = *d*+1.

In conclusion, by adding random noise $Lap\left(\frac{d+1}{\varepsilon_{k}^{0}}\right)$ to the initial clustering center ($sum_{k}^{0}$ and $num_{k}^{0}$) and adding random noise $Lap\left(\frac{d+1}{\varepsilon_{k}^{t}}\right)$ to $num_{k}^{t}$ and $sum_{k}^{t}$ of iteration No. t, the algorithm meets the requirement of *ε*−differential privacy protection. The improved algorithm allocates different privacy budget to different clusters to reduce the number of iterations and improve clustering accuracy.

### The complexity analysis

The algorithm complexity of the traditional k-means clustering algorithm is *O*(T*n*k*d), where T is the number of iterations, n is the number of elements, k is the number of cluster center points, and d is the number of attributes of each element.

In the algorithm of this paper, the key to privacy distribution based on contour coefficients is to calculate the similarity *a* in the cluster and the dissimilarity *b* between clusters. The traditional algorithm complexity for calculating the contour coefficients is *O*(*n*^2^). When the amount of data is large, the calculation speed is significantly reduced. In this paper, the method of calculating the contour coefficient based on the center point is adopted, that is, the intra-cluster similarity *a* is obtained by calculating the distance of each record in the same cluster to the cluster center. Similarly, the inter-cluster dissimilarity *b* is obtained by calculating the minimum value of the distance from all cluster centers of different clusters of this record, thereby reducing the time complexity from *O*(*n*^2^) to *O*(*n*).

In the actual calculation, the calculation of the contour coefficient with complexity *O*(*n*) can be integrated into the clustering process, so the complexity of the algorithm is still *O*(T*n*k*d).

## Experiment findings

### Experimental environment and data

The experimental platform is Intel(R) Core(TM) i5-4460 CPU @ 3.2GHz processor with 4GB memory. The Hadoop cluster environment is deployed on a Linux operating system. The developing software is eclipse4.3 and the algorithm is operated by Java.

The dataset used in the experiment is Dataset “Blood”, “gamma”, “abalone” and “covtype” in UCI Knowledge Discovery Archive database. It is shown in Table 1.

**Table 1:** Datasets used in the experiment.

Dataset	Number of records	Number of characteristics	Type of data
blood	748	5	Real value
abalone	4177	8	Real value
gamma	19200	10	Real value
covtype	581012	54	Real value

The experiment in this paper aims at testing the availability of the algorithm by comparing the clustering effect of the initial algorithm and the improved one after adding random noise.

#### Metrics experiment of the availability of clustering results

Clustering results can be tested by *F*−*measure* [17]. Large value of *F*−*measure* result shows that the clustering results of 2 datasets are close, which means the algorithm has good availability. When the *F*−*measure* result is 1, the clustering results of 2 datasets are the same.

Suppose that *CLUSTER* and *CLUSTER*' represent the 2 clustering results of different clustering algorithms operated on the same dataset *D*. The number of the clusters is *k*. *U*_*i*_ represents clustering collection No. *i*(1≤*i*≤*k*) in *CLUSTER* and *V*_*i*_ represents clustering collection No.i in *CLUSTER*'. |*U*_*i*_| and |*V*_*i*_| represent the number of records in *U*_*i*_ and *V*_*i*_. Suppose that the accuracy of cluster No. *i* is *P*_*i*_ and the recall rate is *R*_*i*_. Then $R_{i}=\frac{\mathrm{cov}er_{i}}{\left|U_{i}\right|}$, $P_{i}=\frac{\mathrm{cov}er_{i}}{\left|V_{i}\right|}$ and $F_{i}=\frac{2R_{i}P_{i}}{R_{i}+P_{i}}$. In the end, each cluster is weighted harmonic averaged. Suppose that *N* is the number of records in the dataset, the availability of clustering result $F-measure=\sum_{U_{i}\in CLUSTER}\frac{\left|U_{i}\right|}{N}F_{i}$.

Suppose that the similarity between the algorithm in Documentary [7] and the classifying result of the dataset without differential privacy protecting noise is *F*−*measure*1 and the similarity between the algorithm in this paper and the classifying result of the dataset without differential privacy protecting noise is *F*−*measure*2. Because of the randomness of Laplace privacy protecting noise, this paper adopted the average value of 10 experiments under the same privacy budget.

**Fig 4:**
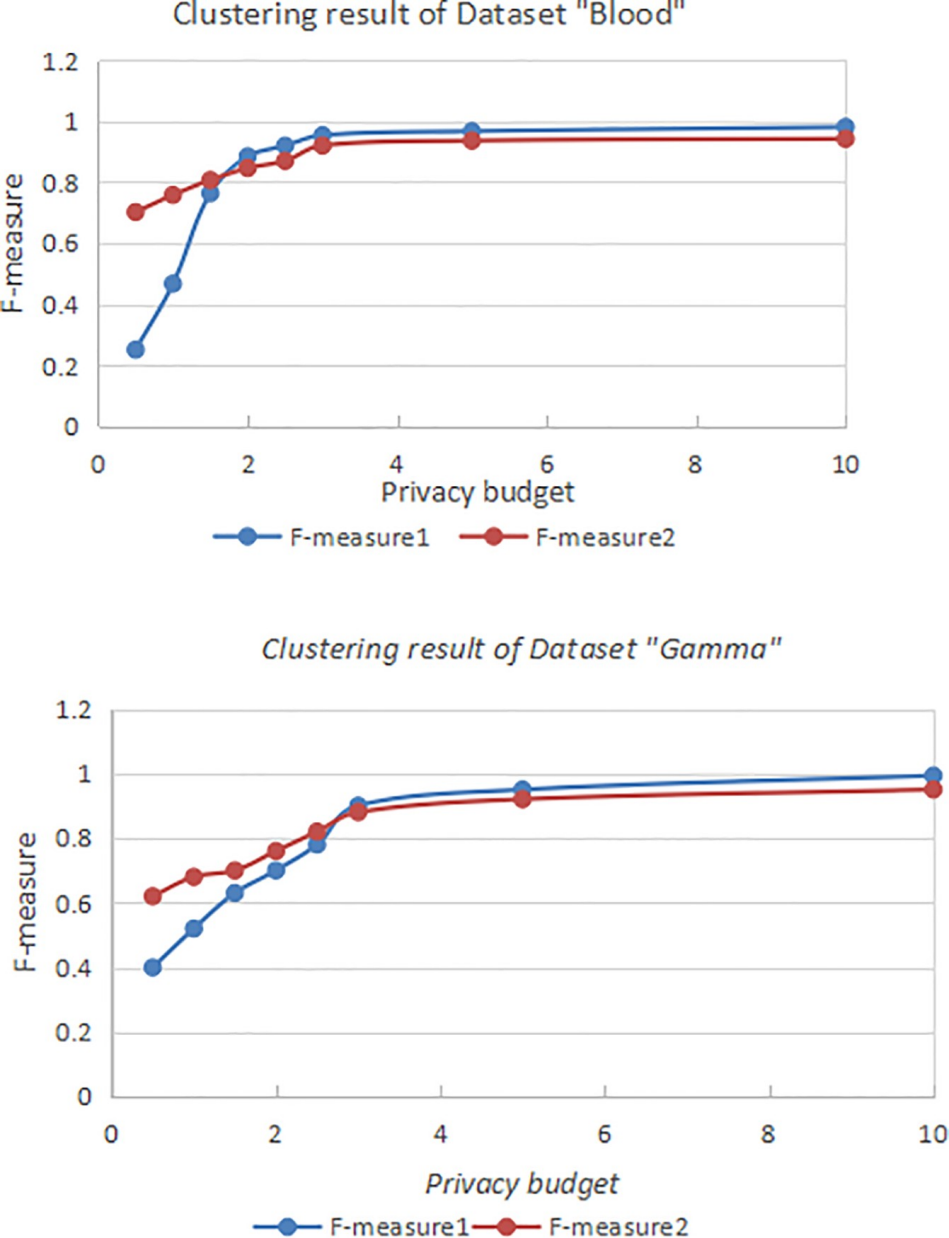
Availability measurement of datasets “Blood” and “Gamma”.

As is shown in Fig 4, when privacy budget *ε* is relatively small, the algorithm proposed in this paper can significantly improve the availability of clustering results. The clustering availability of the algorithm in this paper is not as good as that of the algorithm in Documentary [7]. That is because when *ε* is large, with small random noise, the effect of privacy budget calculated by contour coefficient proposed in this paper on the clustering result of different clusters is small. Under such circumstance, the privacy budget can hardly reflect the features of data. Meanwhile, the contour coefficients in the algorithm decrease some privacy budget, which causes the lower availability of the experimental results as well.

#### Algorithm stability experiment

**Fig 5:**
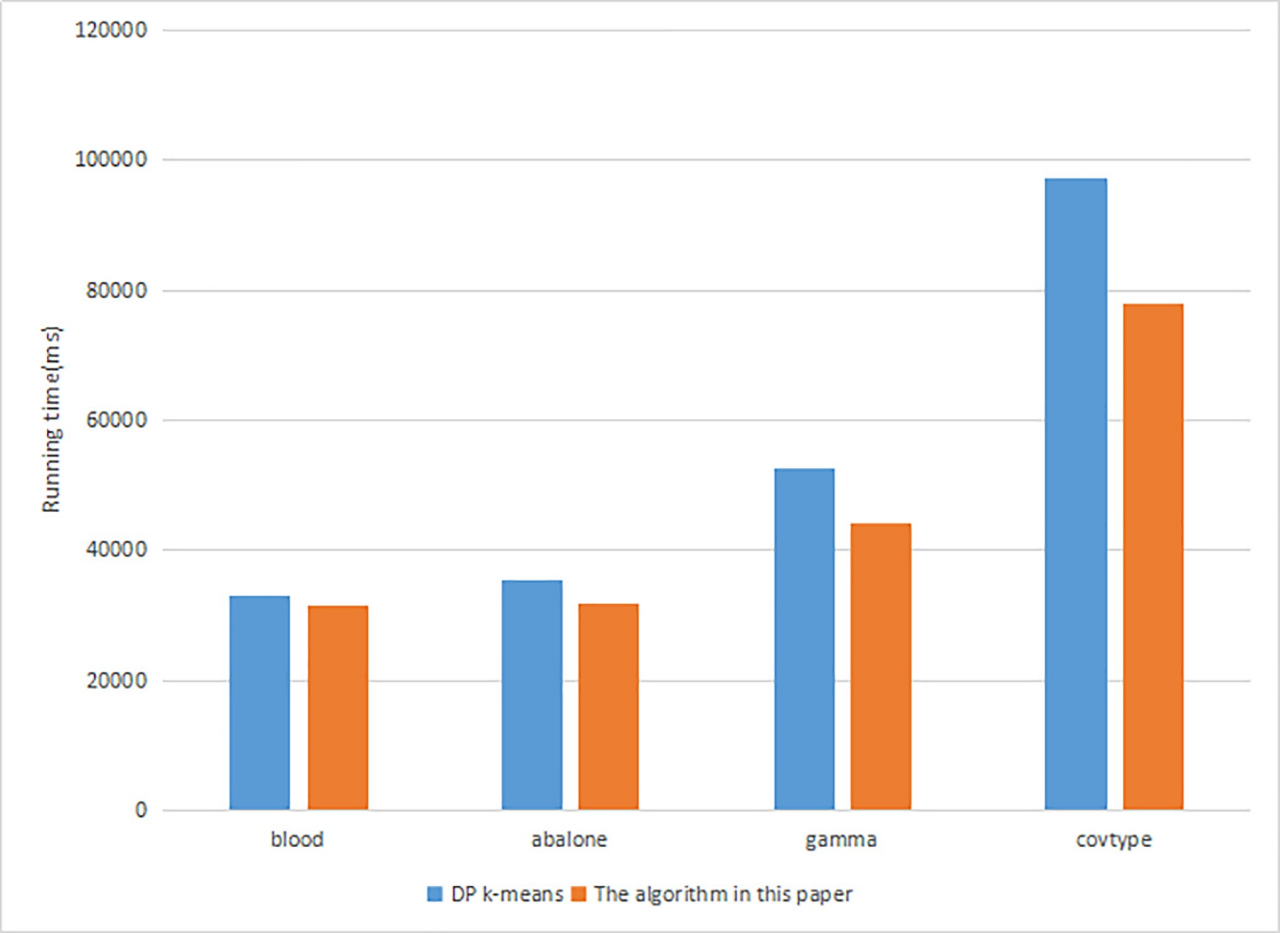
Parallel efficiency comparison in the algorithm in this paper and DP K-means.

In this experiment, four datasets are used. When the amount of data is different, the cluster nodes are the same, and the privacy budget is unchanged, the time spent by the algorithm in this paper and the DP K-means algorithm in a distributed environment is compared. The result is shown in Fig 5.

It can be seen that as the number of records in the dataset increases, the running time of the algorithm gradually increases. The running time of the algorithm in this paper and DP K-means algorithm is reduced due to the algorithm in this paper adds differential privacy based on contour coefficients reduced the number of iterations of the algorithm. The calculation of the contour coefficients in parallel with Map and Reduce processes in MapReduce does not consume more time, so that the algorithm consumption time is reduced in the case of improving the availability of clustering results.

#### Experiment of algorithm efficiency

**Fig 6:**
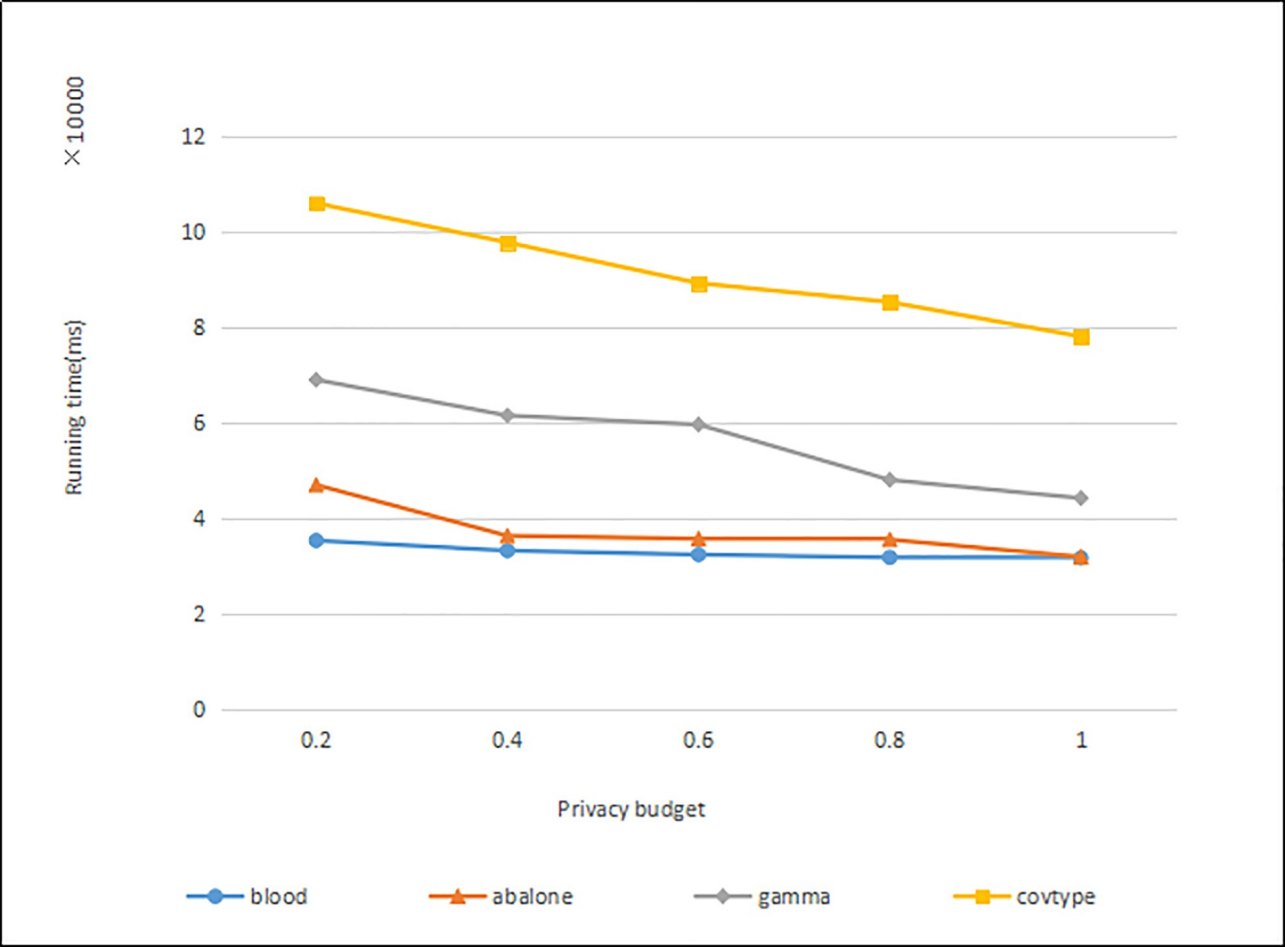
Stability of running time of data sets under different privacy budgets.

The algorithm in this paper mainly aims to improve the usability of clustering results when the privacy budget is small. Therefore, when the privacy budget is small, the data sets of “blood”, “abalone”, “gamma” and “covtype” are used for comparison experiments. The number of nodes is 5, and clustering is performed under different privacy budgets. The experimental results are shown in Fig 6.

It can be seen that the time after the improved parallel algorithm runs on different data sets increases with the privacy budget, and the running time decreases. The larger the privacy budget, the smaller the random noise added by the cluster center point, and the smaller the data is disturbed, so the number of iterations is reduced, and the running time is reduced.

#### Algorithm acceleration ratio analysis

In this experiment, different datasets are used. When the privacy budget is the same, the algorithm acceleration ratio is analyzed when the number of cluster nodes increases.

Through the analysis of the efficiency of the algorithm, it is found that although the improved algorithm adds the calculation of the contour coefficient, the running time of the algorithm is reduced by the design of the algorithm and the design of the contour coefficient in the distributed environment. The performance of the algorithm is measured by the acceleration ratio, which is a ratio of the time consumed by the same task in parallel processing of single nodes and multiple nodes to describe the efficiency of parallel processing. One way to evaluate the acceleration ratio is to keep the amount of data constant and increase the number of nodes in the cluster. Assume that the number of nodes in the cluster is *m*, and the acceleration ratio *S*(*m*) is as follows:

$$S(m)=T_{1}/T_{m}$$ (6)

*T*_1_ is the time required to process data when a single node is used, and *T*_*m*_ is the time when data is processed when the number of nodes is *m*.

**Fig 7:**
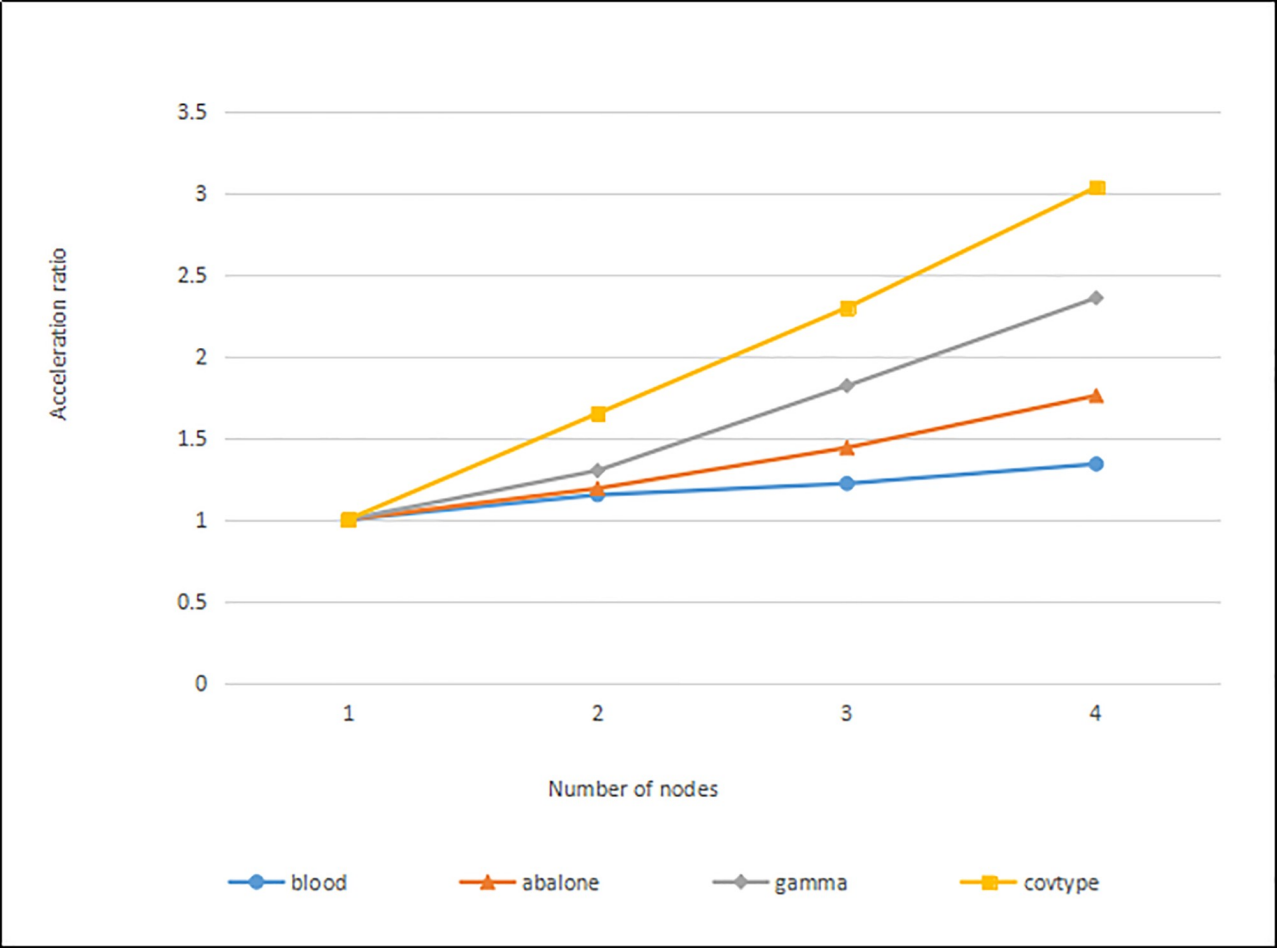
Parallel the algorithm in this paper acceleration ratio.

During the experiment, the data set is processed by using different number of child nodes, and the speedup ratio is calculated. The experimental results are shown in Fig 7.

As shown in the corresponding acceleration ratios in Fig 7, for the "blood" dataset and the "abalone" dataset with smaller data volume, the improvement of efficiency is not obvious when the number of parallel the algorithm in this paper nodes increases. However, for the "gamma" and "covtype" data sets with large data volume, when the number of nodes increases, the acceleration ratio curve of the algorithm is better, and as the size of the data set increases, the acceleration ration performance of the algorithm becomes better. It can be seen that the parallel the algorithm in this paper has better processing power for big data.

## Conclusion

This paper adds differential privacy to K-means clustering algorithm. It evaluates clusters according to contour coefficients and by allocating different privacy budget to different clusters, it adds random noise to different clusters. In this way, the algorithm avoids deviation of the center point caused by too large random noise when privacy budget *ε* is relatively small and solved the problem of unsteady clustering and low accuracy of clustering results. The experiment findings show that the new algorithm, compared to the traditional ones which ignore the cluster features and directly add random noise, provides better clustering results availability, Especially when privacy budget is small, the new algorithm reduces the number of iterations, which is of better realistic significance for privacy protecting clustering algorithms. The next step of the research will be conducted in the following aspects: 1) although the new algorithm improves the availability of the clustering results, when the cluster is small, null clusters may appear because of the random noise. And this may affect the accuracy of experiment.2) the selection of initial center point is not flexible. In this paper, the effect of isolated point on the initial center point is not taken into consideration, which may result in the unsteadiness of clustering results.

## Supporting information

S1 File Experiment code. (ZIP)

## References

[1] Jiang YZ, Chung FL, Wang ST, Deng ZH, Wang J, Qian PJ. Collaborative fuzzy clustering from multiple weighted views[J]. IEEE transactions on cybernetics. 2015; 45(4): 688–701. 10.1109/TCYB.2014.2334595 25069132 10.1109/TCYB.2014.2334595

[2] Zhao X, Evans N, Dugelay JL. A subspace co-training framework for multi-view clustering[J]. Pattern Recognition Letters. 2014; 41: 73–82.

[3] White M, Zhang XH, Schuurmans D. Convex multi-view subspace learning[C]//Advances in Neural Information Processing Systems. 2012; 1673–1681.

[4] Mivule K, Turner C, Ji SY. Towards A Differential Privacy and Utility Preserving Machine Learning Classifier[J]. Procedia Computer Science. 2012; 12(4): 176–181.

[5] He XM, Wang XY, Chen HH, Dong YH. Study on choosing the parameter ε in differential privacy [J]. Journal on Communications. 2015; 36(12); 124–130. [In Chinese]

[6] Li Y, Hao ZF, Wen W, Xie GQ. Research on Differential Privacy Preserving K-means Clustering[J]. Computer Science. 2013; 40(3): 287–290. [In Chinese]

[7] Li HC, Wu XP, Chen Y. k-means clustering method preserving differential privacy in Map Reduce framework [J]. Journal on Communications. 2016; 37(2): 124–130. [in Chinese]

[8] Polatidis N, Georgiadis CK, Pimenidis E, Mouratidis H. Privacy-preserving collaborative recommendations based on random perturbations[J]. Expert Systems with Applications. 2016; 71:18–25.

[9] Yu QY, Luo YL, Chen CM, Ding XT. Outlier-eliminated k-means clustering algorithm based on differential privacy preservation[J]. Applied Intelligence. 2016; 45(4): 1179–1191.

[10] Ren J, Xiong JB, Yao ZQ, Ma R, Lin MWi. DPLK-Means: A Novel Differential Privacy K-Means Mechanism[C]// IEEE Second International Conference on Data Science in Cyberspace. IEEE. 2017; 133–139.

[11] Xiong JB, Ren J, Chen L, Yao ZQ, Lin MW, Wu DP, et al Enhancing privacy and availability for data clustering in intelligent electrical service of IoT[J]. IEEE Internet of Things Journal. 2018.

[12] Xiong P, Zhu TQ, Wang XF. A Survey on Differential Privacy and Applicationgs[J]. Chinese Journal of Computers. 2014; 37(1): 101–122. [In Chinese]

[13] Haeberlen A, Pierce B C, Narayan A. Differential Privacy Under Fire[C]//USENIX Security Symposium. 2011.

[14] Dwork C, McSherry F, Nissim K, Smith A. Calibrating noise to sensitivity in private data analysis//Proceedings of the 3rd Conference on Theory of Cryptography. New York,USA. 2006:265–284.

[15] Zhang XJ, Meng XF. Differential Privacy in Data Publication and Analysis. Chinese Journal of Computers. 2014; 37(4):927–949. [In Chinese]

[16] DWORK C. A Firm Foundation for Private Data Analysis[J]. Communications of the ACM. 2011; 54(1):86–95.

[17] Valentini G, Dietterich T G. Bias-variance Analysis of Support Vector Machines for the Development of SVM-Based Ensemble Methods[J]. Journal of Machine Learning Research. 2004; 5:725–775.

